# Modulation of Deiodinase Types 2 and 3 during Skeletal Muscle Regeneration

**DOI:** 10.3390/metabo12070612

**Published:** 2022-07-01

**Authors:** Ashley Ogawa-Wong, Colleen Carmody, Katherine Le, Rafael Aguiar Marschner, P. Reed Larsen, Ann Marie Zavacki, Simone Magagnin Wajner

**Affiliations:** 1 Division of Endocrinology, Diabetes, and Hypertension, Department of Medicine, Brigham and Women’s Hospital, Boston, MA 02115, USA; anogawa05@gmail.com (A.O.-W.); ccarmody46@gmail.com (C.C.); katherine_le@meei.harvard.edu (K.L.); plarsen@partners.org (P.R.L.); annmarie.zavacki@gmail.com (A.M.Z.); 2 Endocrine Division, Department of Internal Medicine, Hospital de Clínicas de Porto Alegre, Universidade Federal do Rio Grande do Sul, Porto Alegre 9000335, Brazil; rafamarschner@gmail.com

**Keywords:** skeletal muscle, thyroid hormone, deiodinases, muscle injury, FAPs

## Abstract

The muscle stem-cell niche comprises numerous cell types, which coordinate the regeneration process after injury. Thyroid hormones are one of the main factors that regulate genes linked to skeletal muscle. In this way, deiodinase types 2 and 3 are responsible for the fine-tuning regulation of the local T3 amount. Although their expression and activity have already been identified during muscle regeneration, it is of utmost importance to identify the cell type and temporal pattern of expression after injury to thoroughly comprehend their therapeutic potential. Here, we confirmed the expression of Dio2 and Dio3 in the whole tibialis anterior muscle. We identified, on a single-cell basis, that Dio2 is present in paired box 7 (PAX7)-positive cells starting from day 5 after injury. Dio2 is present in platelet derived growth factor subunit A (PDGFA)-expressing fibro-adipogenic progenitor cells between days 7 and 14 after injury. Dio3 is detected in myogenic differentiation (MYOD)-positive stem cells and in macrophages immediately post injury and thereafter. Interestingly, Dio2 and Dio3 RNA do not appear to be present in the same type of cell throughout the process. These results provide further insight into previously unseen aspects of the crosstalk and synchronized regulation of T3 in injured muscle mediated by deiodinases. The set of findings described here further define the role of deiodinases in muscle repair, shedding light on potential new forms of treatment for sarcopenia and other muscular diseases.

## 1. Introduction

Tissue regeneration allows damaged tissues to repair and remodel upon injury. Skeletal muscle is composed of multinucleated mature muscle cells (myofibers), a resident pool of muscle stem cells (MuSCs, also called muscle satellite cells), and other populations such as fibro-adipogenic progenitors (FAPs), endothelial cells, tenocytes, and resident immune cells [1,2,3]. Mammalian skeletal muscle exhibits the capacity for extensive regeneration in response to injury [4]. It is critical to understand the cell types and processes that mediate skeletal muscle healing to improve regenerative efficiency while limiting scar formation. Muscle repair, which mainly depends on satellite cells, counteracts skeletal muscle loss and supplies new myofibers [5,6].

Muscle is a major target of TH action [7,8]. T3 potentiates satellite cell differentiation and muscle regeneration upon injury [9,10]. THs also regulates oxygen consumption, fiber composition, calcium mobilization, and glucose uptake [11,12]. While the role of thyroid hormones is well established as part of muscle recovery after injury [7], the precise cells in which types 2 and 3 deiodinases are located, as well as the time course of their expression during regeneration, are still not well defined. Type 2 deiodinase (Dio2) converts thyroxine to 3,3,5-triiodothyronine (T3), while type 3 deiodinase (Dio3) inactivates T3, thus fine-tuning intracellular T3 regulation. Although Dio2 expression is low in normal muscle, its expression and activity, as measured in mixed fiber-type skeletal muscle homogenates, increase after injury to provide additional T3 for differentiation [13,14]. Indeed, many genes in muscle are TH-responsive, and intracellular concentrations of THs must be tightly regulated in satellite cells during regeneration [7]. The increased Dio2 in stem cells during skeletal muscle regeneration leads to T3-dependent potentiation of differentiation [15,16,17]. In contrast to Dio2, Dio3 activity and intracellular T3 concentration have been reported to increase and decrease, respectively, in the skeletal muscle of patients during hospitalization in an intensive care unit, and in animal models [18,19,20]. Thus, T3 might decrease in a *DIO3*-dependent manner in the skeletal muscle of individuals with severe health problems [19].

Muscle satellite cells are necessary for long-term maintenance of muscle functionality. Previous studies have shown that, in non-injured muscle, other than myofibers, FAPs correspond to 62%, satellite cells correspond to 3.1%, and immune cells correspond to 7% of the total cell population in muscle tissue [3]. The muscle healing process leads to many changes after injury and is a very dynamic process. Immediately after injury, immune cells account for 87% of the local cell population, while FAPs drop to 10% [3]. Within this system, the timing of Dio2/Dio3 expression to modulate THs in specific cell types is a crucial part of normal skeletal muscle repair after injury.

Considering the importance of T3 in skeletal muscle recover after injury, we designed a study to dissect, on a single-cell basis, the exact location and timing of expression of both Dio2 and Dio3 in MuSCs cells, FAPs, and immune cells, part of the regeneration process essential for skeletal muscle function. While the activity of D2 and D3 in the whole muscle is well known, the aim of this work was to identify the precise time and location of Dio2 and Dio3 inside the different muscle cell compartments during regeneration.

## 2. Results

### 2.1. Dio2 and Dio3 Are Expressed throughout the Regeneration Process

**Figure 1:**
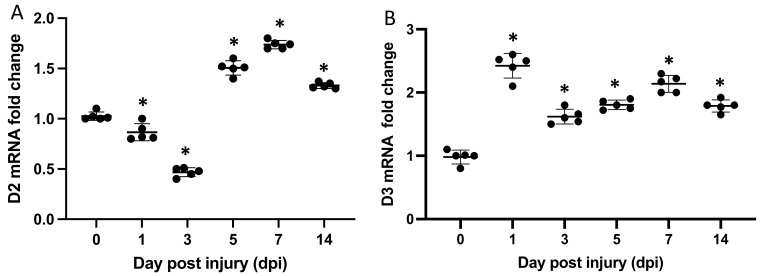
Time course of mRNA levels of D2 and D3 according to real-time PCR analysis (*n* = 5) in whole muscle tissue. (**A**) mRNA levels of Dio2 during the post-injury recovery process. The highest expression occurred between days 5 and 7. (**B**) mRNA levels of Dio3 during the post-injury recovery process. Dio3 expression peaked on day 1 after injury and remained stable thereafter. * *p* = 0.0001. Data are expressed as the mean ± SEM of at least three independent experiments (*n* = 5 for each experiment).

We first determined the expression of Dio2 and Dio3 in the whole TA (tibialis anterior) muscle and observed that the highest expression in injured tissue occurred from day 5 to day 7 for Dio2, after a nadir on day 3 (Figure 1A). Interestingly, the expression of Dio3 in the whole TA peaked on day 1 and remained stable thereafter (Figure 1B).

### 2.2. Dio2 Is Located in Muscle Satellite Pax7^+^ Cells

**Figure 2:**
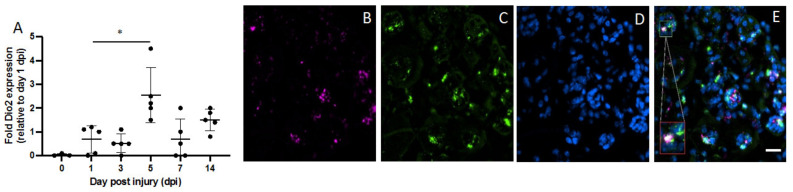
Dio2 and its presence in stem cells. (**A**) Augmented expression of Dio2 in isolated Pax7^+^ stem cells between days 1 and 14 after injury measured by RT-PCR. In situ hybridization on day 5 after injury showing Dio2 mRNA stained in pink (**B**) and Pax7^+^ cells in green (**C**) and DAPI in blue (**D**). (**E**) Colocalization of Dio2 in Pax7^+^ cells. * *p* = 0.0001. Data are expressed as the mean ± SEM. Images are representative of injury in one of five different mice analyzed (*n* = 5). Scale bar = 50 µm.

Next, we aimed to identify the precise Dio2-expressing cell type and define how expression changes over time. Pax7^+^ muscle stem cells were the cell type mainly expressing Dio2 (Figure 2E). Expression occurred from day 5 and was still present 14 days after injury, peaking on day 5 after injury.

**Figure 3:**
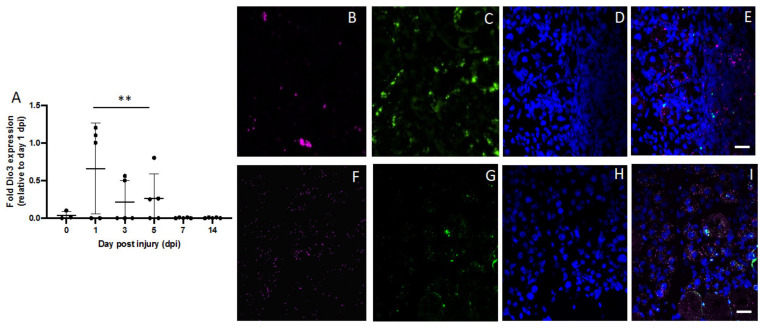
Dio3 and its expression in Pax7^+^ and MyoD^+^ stem cells. (**A**) Dio3 expression between days 0 and 14 after injury in Pax7^+^ stem cells as measured by RT-PCR. (**B**,**C**) In situ hybridization showing expression of Dio3 (pink) and isolated Pax7^+^ cells (green) and DAPI (**D**) on day 5 post injury. (**E**) Dio3 expression in only a few Pax7^+^ cells. (**F**,**G**) In situ hybridization showing Dio3 (pink) expression and MyoD^+^ cells (green), whereas DAPI is blue (**H**). (**I**) Merged image showing Dio3 expression on day 5 in MyoD^+^ cells. ** *p* = 0.003. Data are expressed as the mean ± SEM of at least three independent experiments. Images are representative of injury on day 5 in one of five different mice analyzed (*n* = 5). Scale bar = 50 µm.

When we assessed the expression of Dio3 in the whole muscle, we could see a sustained expression throughout the recovery process (Figure 1B). We then evaluated Dio3 expression in stem cells and observed that, in this cell type, Dio3 was present from days 1 to 5 at highly variable levels (Figure 3A). Next, we analyzed in which cell type Dio3 may possibly be located, and we detected its presence in low levels (Figure 3B) in Pax7^+^ cells (Figure 3C,E). Subsequently, we examined Myod^+^ cells and detected high Dio3 expression in these cells compared to Pax7^+^ cells on day 5 post injury.

### 2.3. Fibroadipogenic Progenitors (FAPs) Express Dio2 and Dio3 in a Temporal Fashion

**Figure 4:**
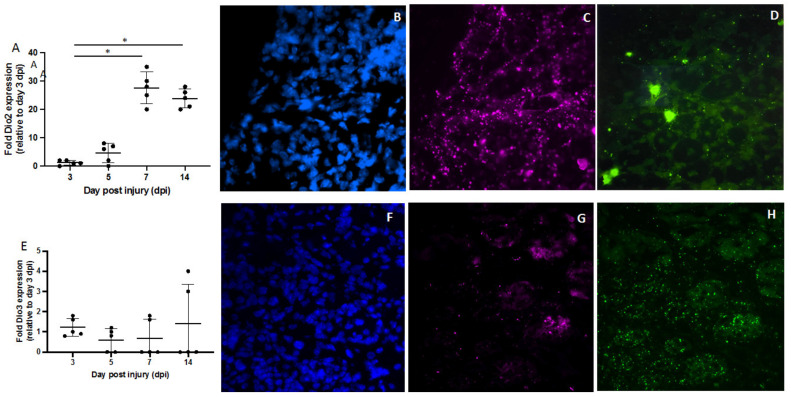
Expression of Dio2 and Dio3 in PDGFA-positive cells. (**A**) Higher expression of Dio2 occurred later in muscle recovery, from day 7 to day 14 (RT PCR, * *p* = 0.0001). (**B**) DAPI in blue, (**C**) PDGFA-positive cells in pink (**D**) Dio2 expression in green on day 7 after injury. (**E**) RT-PCR showing that the highest Dio3 expression occurred on day 14. (**F**) DAPI in blue, (**G**) PDGFA-positive cells in pink on day 14 and (**H**) Dio3 in the same cells in green. Data are expressed as the mean ± SEM of at least three independent experiments. Images are representative of injury on day 7 for Dio2 and day 14 for Dio3 in one of five different mice analyzed (*n* = 5). Scale bar = 50 µm.

Then, we evaluated Dio2 expression in isolated FAPs and observed that the highest expression of Dio2 occurred between days 7 and 14, as shown in Figure 4A. The injured muscle showed a particularly high single-cell staining of the FAP-specific marker PDGFA (platelet-derived growth factor subunit A; see Figure 4C) on day 7, which colocalized with high Dio2 expression (Figure 4D). Interestingly, Dio3 expression increased on day 14, whereas Dio2 expression decreased (Figure 4E,G,H).

**Figure 5:**
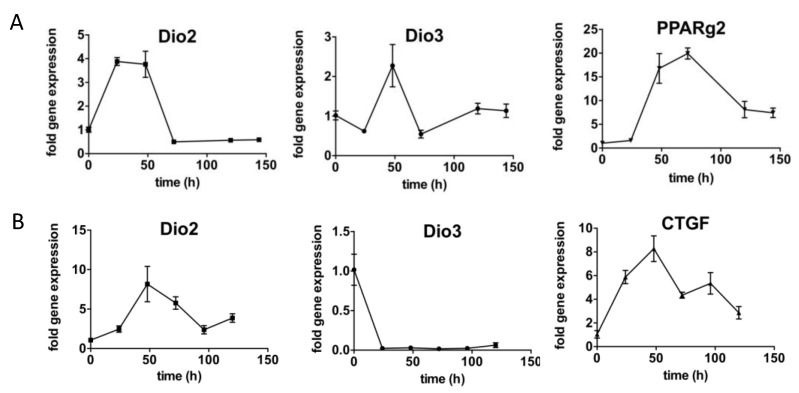
Primary culture of FAP cells differentiated to adipocytes. (**A**) Presence of PPARg2, confirming adipocytes, showing the expression of Dio2 and higher expression of Dio3. (**B**) Presence of CTGF, confirming the presence of collagen and indicating differentiation into myofibroblasts, show a substantially higher expression of Dio2 with no expression of Dio3.

A bilinear trajectory of FAP populations was described, resulting in populations diverging into myofibroblasts or adipocytes, strongly influencing the trajectory of muscle repair [1,8]. We then assessed Dio2 and Dio3 expression in a primary cell culture model enriched for FAPs that was differentiated either into adipocytes (Figure 5A) or myofibroblasts (Figure 5B). Interestingly, a completely different trajectory of both enzymes can be observed through time. When cells were differentiated to adipocytes, Dio2 peaked on the second day and was not detected after that, whereas Dio3 peaked as Dio2 expression began to decline (Figure 5A). When cells differentiated to myofibroblasts, Dio2 expression peaked after 2 days, whereas Dio3 expression rapidly declined (Figure 5B).

### 2.4. Type 3 Deiodinase Is Mainly Expressed in Macrophages during the Muscle Recovery Process

**Figure 6:**
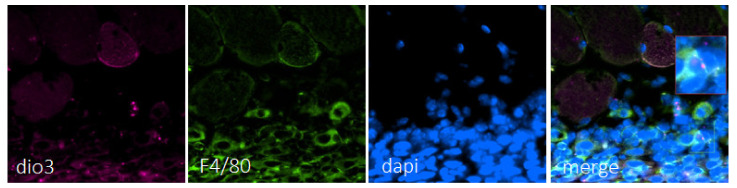
Dio3 mRNA in pink was predominately expressed in macrophages (F4/80 immunostaining, green; nucleus DAPI staining, blue). The merged panel shows that Dio3 mRNA in pink was present in macrophages. Images are representative of in situ hybridization for Dio3 in injured muscle on day 1 while F4/80-stained images are representative of macrophages in one of five different mice analyzed (*n* = 5). Scale bar = 50 µm.

We sought to observe deiodinases in immune cells during the recovery process. While Dio2 was not detected in this type of cell, Dio3 was confirmed to be in macrophages during the recovery process, starting on day 1 (Figure 6).

## 3. Discussion

This descriptive study determined the location of Dio2 and Dio3 at the single-cell level in different skeletal muscle cell types during the process of muscle recovery. The objective was to further clarify the coordination of Dio2 with Dio3 in regenerating muscles and define the precise cell types that express these enzymes. We observed that Dio2 and Dio3 appeared to be not expressed in the same cell type at the same time; Dio2 expression occurred mostly in Pax7^+^ cells, while that of Dio3 occurred in MyoD^+^ cells later in the process. After injury, PDGFA^+^ FAPs mostly expressed Dio2 in high levels on day 7 in the process of recovery. In contrast, type 3 deiodinase was primarily expressed in macrophages. Changes in this expression pattern suggest that local changes in T3 content can alter the regeneration process. The initial process that precedes recovery is the local immune response, which is led by macrophages mediating clearance of damaged tissue after injury [2]. Dio3 expression is concentrated in macrophages, suggesting that a strict level of T3 control is required to clear the necrotic tissue during the muscle repair process [18].

The activation of stem cells occurs very early in the process and is required for maintenance and regeneration of skeletal muscle [21,22]. At the same time, during the first days after injury, FAPs undergo massive expansion, as observed here and by other authors [23]. In addition, FAPs are critical during regeneration to sustain satellite cells [24]. This affirmation is in total agreement with the results observed here through coordination of deiodinase types 2 and 3, indicating that T3 production regulated by Dio2 begins in Pax7^+^ cells and then becomes predominant in FAPs, potentially acting in a paracrine mechanism to regulate the regeneration process. Parallel to this process, Dio3 is expressed in macrophages and Myod^+^ cells, as well as in FAPs later on in the regeneration process. Notably, the Dio3 signal found here in Pax7^+^ cells could be interpreted as much lower than expected on the basis of previous data [18]. However, here, we aimed to see the enzyme behavior in specific cells while preserving the whole tissue architecture; in other studies, Dio3 was assessed using a different approach to Pax7^+^ cultured cells. However, even considering the different approaches used in these studies, both sets of results are consistent regarding the Dio3 expression pattern of injured TA on day 5 of recovery under the same cardiotoxin injection model.

One important Dio2 characteristic that must be discussed here is its post-transcriptional regulation. T4 to T3 conversion by the Dio2 enzyme can result in ubiquitination and degradation of the protein, acting as a potent mechanism to regulate T3 production [25,26]. Since our studies were at the mRNA level, determining the regulation of T3 levels in cell types via these mechanisms was not the aim here and could not be evaluated. However, it is relevant to note that Dio2 mRNA must first be expressed for post-translational regulatory mechanisms to be relevant afterward.

On the basis of the expression of PDGFA, FAPs may mediate muscle remodeling at both the early and late regenerative stages since FAPs are the main producer of the extracellular matrix required for normal regeneration [11,12]. However, if this process is deregulated, these cells can turn into adipocytes, impairing the regeneration process. Interestingly, we demonstrated here that this fate might be regulated, at least in part, by Dio2 and Dio3. Of note, Dio3 is increased not only by growth factors such as PDGF but also by cytokines, especially IL6 [16]. Dio3 expression in FAPs and macrophages in an otherwise normal process of healing [27], under conditions of a prolonged stimulus, could explain the Dio3 overexpression observed on skeletal muscle in a state of disease [19]. The results shown here lead to new insights into our understanding of why some patients with a different basis of disease develop skeletal muscle injury such as sarcopenia and have a different time course of recovery. In this regard, it is important to emphasize that FAPs regulated by IL-6 could have two opposite effects on muscle. In a normal regeneration process, IL-6 acts as a promyogenic molecule [28]. However prolonged inflammatory situations, as seen in several diseases and more recently the SARS-CoV-19 inflammatory disease, present sustained high levels of IL-6, where FAP dysregulation could promote muscle atrophy and denervation muscle wasting with fibrosis. This compelling observation drives us to consider new mechanisms that must be further investigated.

The set of results presented here shed light on the muscle regeneration process as seen in a detailed single-cell approach with the spatial tissue component preserved. Future studies will be important to see how the different expression patterns of Dio2 and Dio3 shown here change in acute and prolonged pathogenic situations. This knowledge will provide different approaches to facilitate recovery in patients with muscle injury.

## 4. Materials and Methods

### 4.1. Animals

All animal studies were approved by the Institutional Animal Care and Use Committee of Brigham and Women’s Hospital (Number # A4752-01). Mice were male and 8–14 weeks of age. The tibialis anterior (TA) muscle of mice was injured by injection of 20 µL of 10 µM cardiotoxin as previously described, and TA was collected on days 1, 3, 5, 7, and 14 post injury [4].

### 4.2. Fluorescence-Activated Cell Sorting

Muscle stem cells were isolated from TA as previously described [13]. Briefly, the TA of mice was collected and digested in collagenase II (75 U/mL), followed by digestion in dispase II (2.4 U/mL)/collagenase D (1.5 U/mL) (Sigma-Aldrich, St. Louis, MO, USA). After passing through a 40 µm cell strainer, cells were stained for CD45 (1:500, phycoerythrin, 103106; Biolegend, San Diego, CA, USA), CD34 (1:200, fluorescein isothiocyanate, 560238; BD Biosciences, San Jose, CA, USA), CD31(1:500, PacBlue, 102422; Biolegend), Sca1 (1:200, APC, 122512; Biolegend), and a7-integrin (1:200, AF750, 75-0010-05; AbLab, Vancouver, BC, Canada). FACS was performed using a BD FACSAria II flow cytometer (BD Biosciences), and data were analyzed using FlowJo software (FlowJo LLC, Ashland, OR, USA). Muscle stem cells were identified as linSca1-a7 [21,22].

### 4.3. Cell Culture

To isolate satellite cells from intact adult skeletal muscle, we mechanically and enzymatically dissociated a single-cell suspension, as described. The muscle cells were resuspended in proliferation medium and plated on collagen type I-coated flasks. Freshly isolated cells were cultured for 3 days in high-glucose DMEM (Sigma-Aldrich, St. Louis, MO, USA) containing 10% FCS and penicillin-streptomycin on eight-well Lab-Tek Chamber slides (Nalge Nunc International, Rochester, NY, USA). For fibrotic differentiation, the cells were additionally cultured with TGFβ1–TGFβ3 for 3–5 days (Sigma-Aldrich, St. Louis, MO, USA). For adipogenic differentiation, the cells were additionally cultured in adipogenic induction medium (Cambrex Bioscience, Walkersville, MD, USA) for 3 days and then cultured for 1 day in adipogenic maintenance medium (Cambrex Bioscience). This procedure was repeated three times. Then cells were maintained for 5 more days in the adipogenic maintenance medium as described previously [29,30].

### 4.4. RNAscope In Situ Hybridization

TA and soleus muscles were frozen in dry ice-cooled isopentane and embedded in Tissue-Tek O.C.T (Sakura, Torrance, CA, USA); 7 µm sections were cut using a cryostat. The in situ hybridization of PDGF, PAX7, Dio2, and Dio3 mRNAs was performed in the different sections of uninjured (*n* = 5) and injured (*n* = 5) utilizing the RNAscope technique (Advanced Cell Diagnostics, BioTechne Corporation, Newark, CA, USA) following the manufacturer’s suggested procedures. We used the RNAscope Dio2, Dio3, PDGFA, PAX7, and MYOD probes (catalog numbers 479331, 561641, 411361, 432771, and 316081, respectively) and the Akoya Detection kit to obtain the fluorescence results, as indicated by the manufacturer. As negative and positive controls, we used the material supplied by the manufacturer, which had the expected negative or positive results. Tissue sections were mounted with DAPI Fluoromount-G (Catalog # 0100-20, Southern Biotech, Birmingham, AL, USA). Samples were imaged using a Nikon H600L microscope with a 40× objective (Nikon, Tokyo, Japan). The images shown here are illustrative of the complete sections analyzed. H&E stains were performed to verify the quality of the sample before each experiment.

### 4.5. RNA Extraction and Real-Time Quantitative Polymerase Chain Reaction

RNA was extracted from fluorescence-activated cell sorting (FACS, FAPs)-purified muscle stem cells and whole muscle using Trizol (Thermo Fisher Scientific, Waltham, MA, USA) according to the manufacturer’s instructions. Primers were synthesized by Invitrogen, and mouse cyclophilin-A was used as the internal reference gene. All primer sequences can be found in [5] and are listed below. Reverse transcription was performed using the high-capacity cDNA reverse transcription kit (Thermo Fisher Scientific), according to the manufacturer’s instructions. Gene expression was detected using SYBR Select Master Mix (Applied Biosystems, Waltham, MA, USA) on the LightCycler II (Roche, Burlington, MA, USA). The delta delta Ct method was used to calculate the fold change in gene expression compared with the uninjured WT control of at least five animals from each group. Experiments were repeated at least three times. Primers: Pax7 F: TCC CCC TGG AAG TGT CCA, R: TGG GAA ACA CGG AGC TGA; PGC-1α F: TGA GTA ACC GGA GGC ATT CTC T, R: TGA GGA CCG CTA GCA AGT TTG; Dio2 F: CCT CCT AGA TGC CTA CAA ACAG, R: TGA TTC AGG ATT GGA GAC GTG; MyoD F: ACC CAG GAA CTG GGA ATG GA, R: AAG TCG TCT GCT GTC TCA AA.

### 4.6. Statistical Analysis

Statistical analysis was performed using GraphPad Prism 9 software (Graphpad, San Diego, CA, USA), with *p* < 0.05 being considered statistically significant. The results are shown as the means ± SD throughout. When the samples exhibited a normal distribution, differences between samples were assessed using the Student’s two-tailed *t*-test for independent samples or ANOVA and Dunnett’s test. When the distribution of samples was not normal, appropriated tests were applied. Relative mRNA levels (in which the control sample was arbitrarily set as 1) are reported as results of real-time PCR, in which the expression of cyclophilin A served as the housekeeping gene. In all experiments, differences were considered significant when *p* was less than 0.05, as indicated by asterisks throughout.

## Data Availability

The data presented in this study are available on request from the corresponding author. The data are not publicly available due to that some data is still unpublished.
